# CD52 and OXPHOS—potential targets in ibrutinib-treated mantle cell lymphoma

**DOI:** 10.1038/s41420-022-01289-7

**Published:** 2022-12-31

**Authors:** Viktoria Fuhr, Shanice Heidenreich, Mugdha Srivastava, Angela Riedel, Johannes Düll, Elena Gerhard-Hartmann, Andreas Rosenwald, Hilka Rauert-Wunderlich

**Affiliations:** 1 Institute of Pathology, University of Würzburg and Comprehensive Cancer Center (CCC) Mainfranken, Würzburg, Germany; 2 Core Unit Systems Medicine, University of Würzburg, Würzburg, Germany; 3 Mildred Scheel Early Career Center (MSNZ), University Hospital of Würzburg, Würzburg, Germany; 4 Department of Internal Medicine 2, University Hospital of Würzburg, Würzburg, Germany

**Keywords:** Cell death, Cancer metabolism, Targeted therapies, Target validation

## Abstract

Altered features of tumor cells acquired across therapy can result in the survival of treatment-resistant clones that may cause minimal residual disease (MRD). Despite the efficacy of ibrutinib in treating relapsed/refractory mantle cell lymphoma, the obstacle of residual cells contributes to relapses of this mature B-cell neoplasm, and the disease remains incurable. RNA-seq analysis of an ibrutinib-sensitive mantle cell lymphoma cell line following ibrutinib incubation of up to 4 d, corroborated our previously postulated resistance mechanism of a metabolic switch to reliance on oxidative phosphorylation (OXPHOS) in surviving cells. Besides, we had shown that treatment-persisting cells were characterized by increased CD52 expression. Therefore, we hypothesized that combining ibrutinib with another agent targeting these potential escape mechanisms could minimize the risk of survival of ibrutinib-resistant cells. Concomitant use of ibrutinib with OXPHOS-inhibitor IACS-010759 increased toxicity compared to ibrutinib alone. Targeting CD52 was even more efficient, as addition of CD52 mAb in combination with human serum following ibrutinib pretreatment led to rapid complement-dependent-cytotoxicity in an ibrutinib-sensitive cell line. In primary mantle cell lymphoma cells, a higher toxic effect with CD52 mAb was obtained, when cells were pretreated with ibrutinib, but only in an ibrutinib-sensitive cohort. Given the challenge of treating multi-resistant mantle cell lymphoma patients, this work highlights the potential use of anti-CD52 therapy as consolidation after ibrutinib treatment in patients who responded to the BTK inhibitor to achieve MRD negativity and prolong progression-free survival.

## Introduction

Mantle cell lymphoma (MCL) is a rare mature B-cell neoplasm and represents a heterogeneous disease in view of clinical and biological features [1]. The tumor cells typically harbor the t(11;14)(q13;q32) translocation causing cyclin D1 overexpression [2]. While the classical MCL subtype is characterized by nodal localization, a leukemic non-nodal variant with more indolent behavior is distinguished [1]. However, most patients have peripheral blood and bone marrow involvement at clinical presentation regardless of the subtype [3, 4]. Despite improved prognosis since the use of high-dose cytarabine and rituximab-based immunochemotherapy with autologous stem cell transplantation, MCL remains incurable [5]. Due to tumor cell dependence on B-cell receptor signaling and NF-κB signaling, the inhibition of the Bruton tyrosine kinase by ibrutinib achieves high efficacy in relapsed/refractory MCL [6]. However, residual cells that have adapted to ibrutinib treatment may cause minimal residual disease and eventually relapse [4]. Consequently, understanding the mechanisms of acquired resistance driven by ibrutinib is of immediate clinical relevance for the development of effective add-on or subsequent therapies.

Previously, we elucidated early transcriptomic alterations in cells persisting on 48 h (h) ibrutinib treatment by scRNA-seq using REC-1, an ibrutinib-sensitive mantle cell lymphoma cell line [7]. Surviving cells upregulated *CD52* and separated into two distinct metabolic subgroups. Further experiments confirmed higher CD52 surface density and suggested a link between survival and increased dependence on oxidative phosphorylation (OXPHOS).

Here, we performed a bulk RNA-seq analysis to decipher the features of MCL cells that were finally adapted to ibrutinib by prolonging the treatment of REC-1 cells up to 4 days (d). As RNA-seq data corroborated increased activity of oxidative phosphorylation in resisting cells, we focused on OXPHOS and the previously postulated CD52 as potential targets [7].

Cancer cells with a high proliferation rate rely on balanced cellular metabolism to meet energy demands and to cope with distinct microenvironments and therapeutic pressure [8]. Reprogramming cellular metabolism is therefore a cancer hallmark and a crucial player in resistance dynamics [9, 10]. Besides Warburg’s theory of cancer cell’s reliance on glycolysis independent of oxygen availability, OXPHOS has emerged as a potential target in cancer stem cells of chronic myeloid leukemia and breast cancer, in chemotherapy-resistant NSCLC, acute myeloid leukemia, melanoma, and other entities [11–17]. In MCL, ibrutinib treatment impaired glycolysis in sensitive cells, whereas metabolic reprogramming to oxidative phosphorylation (OXPHOS) was associated with resistance to the BTK inhibitor [18, 19]. The small molecule IACS-010759 (IACS) was developed to inhibit OXPHOS by blocking complex I of the mitochondrial electron transport chain (ETC) and is currently being evaluated in phase I clinical trials (NCT02882321, NCT03291938) [20].

Targeting surface antigens of malignant B cells with specific monoclonal antibodies (mAb) has revolutionized cancer therapy [21]. CD52 is a small surface glycoprotein mainly found on mature immune cells like lymphocytes, and to a lesser extent on stem and progenitor cells [22–24]. The molecule may be involved in transduction of proliferative and activating signals and in transendothelial migration of T cells, anti-adhesion, or may exert an inhibitory effect on BCR signaling [25–30]. Alemtuzumab is a humanized IgG1 kappa antibody that is approved for relapsing-remitting multiple sclerosis and had been used in chronic lymphocytic leukemia (CLL) [31–33]. After binding to CD52 on target cells, the antibody can induce antibody-dependent cellular cytoxicity (ADCC) and complement-dependent-cytotoxicity (CDC), but direct cytotoxicity is controversial [34–37].

In this work, the impact of ibrutinib plus OXPHOS inhibition by IACS-010759 on proliferation, apoptosis, and metabolism of MCL cell lines were studied. In addition, we investigated the efficacy of treatment with ibrutinib and CD52 mAb combined with active complement on viability and proliferation of MCL cell lines and primary tumor cells from ten MCL patients.

## Results

### REC-1 show stable transcriptome across 2 to 4 d ibrutinib treatment

**Fig. 1:**
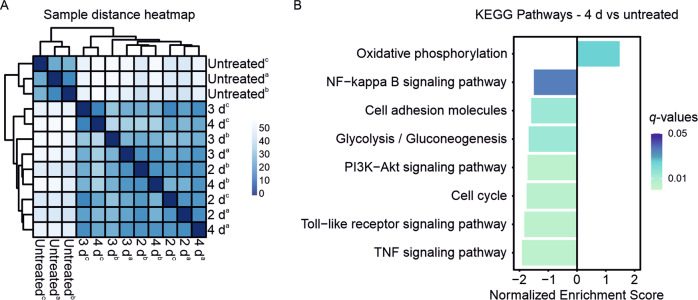
RNA-seq of REC-1 during ibrutinib treatment. **A** Heatmap showing sample-to-sample distances of untreated and ibrutinib-treated (2 d, 3 d, 4 d) samples of three independent replicates (a, b, c) based on the variance stabilizing transformed values. **B** Selected significant pathways (|log2FC | ≥ 1, *q*-value < 0.05) of 4 d ibrutinib-treated REC-1 (vs untreated) from Kyoto Encyclopedia of Genes and Genomes (KEGG) pathway gene set enrichment analysis (GSEA).

The bulk RNA-seq analysis assessed alterations in transcriptome of ibrutinib-sensitive mantle cell lymphoma cells across extended ibrutinib incubation. Therefore, REC-1 cells were left untreated or were treated for 2 d, 3 d, and 4 d with 400 nM ibrutinib. Viable cells were isolated to capture the gene expression profile of the ibrutinib-surviving cell population. While 385 genes were up- and 633 genes were downregulated between 2 d and untreated, only eight genes remained up- and four genes downregulated in 2 d compared with 4 d (|log2FC| ≥ 1, padj < 0.05). The similarity of 2 d, 3 d, and 4 d treatment is shown by the sample distance heatmap Fig. 1A). The untreated cluster apart from the treated samples. However, there is no differentiation between 2 d, 3 d, or 4 d incubation with ibrutinib.

Differentially expressed genes (DEGs) between 4 d and untreated were assessed for gene set enrichment of Kyoto Encyclopedia of Genes and Genomes (KEGG) pathways. Positive enrichment scores were detected for 16 gene sets and negative enrichment was assigned to 82 pathways (|log2FC| ≥ 1, *q*-value < 0.05; Supplementary Table 1). After 4 d of ibrutinib, downstream signaling of the B-cell receptor such as NF-ĸB (NES = −1.50, *q*-value = 0.036), PI3K-Akt (NES = −1.72, *q*-value = 0.005), Toll-like receptor (NES = −1.83, *q*-value = 0.005), and TNF signaling (NES = −1.92, *q*-value = 0.005) were weakened (Fig. 1B, Supplementary Fig. 1). Besides reduced cell cycle activity (NES = −1.76, *q*-value = 0.005), expression of cell adhesion molecules (NES = −1.60, *q*-value = 0.014) decreased. While glycolysis/gluconeogenesis (NES = −1.68, *q*-value = 0.014) was impaired, ibrutinib caused enhanced oxidative phosphorylation (OXPHOS, NES = 1.48, *q*-value = 0.025).

### Combination of ibrutinib and OXPHOS inhibitor IACS-010759 is highly toxic to ibrutinib-sensitive cells

**Fig. 2:**
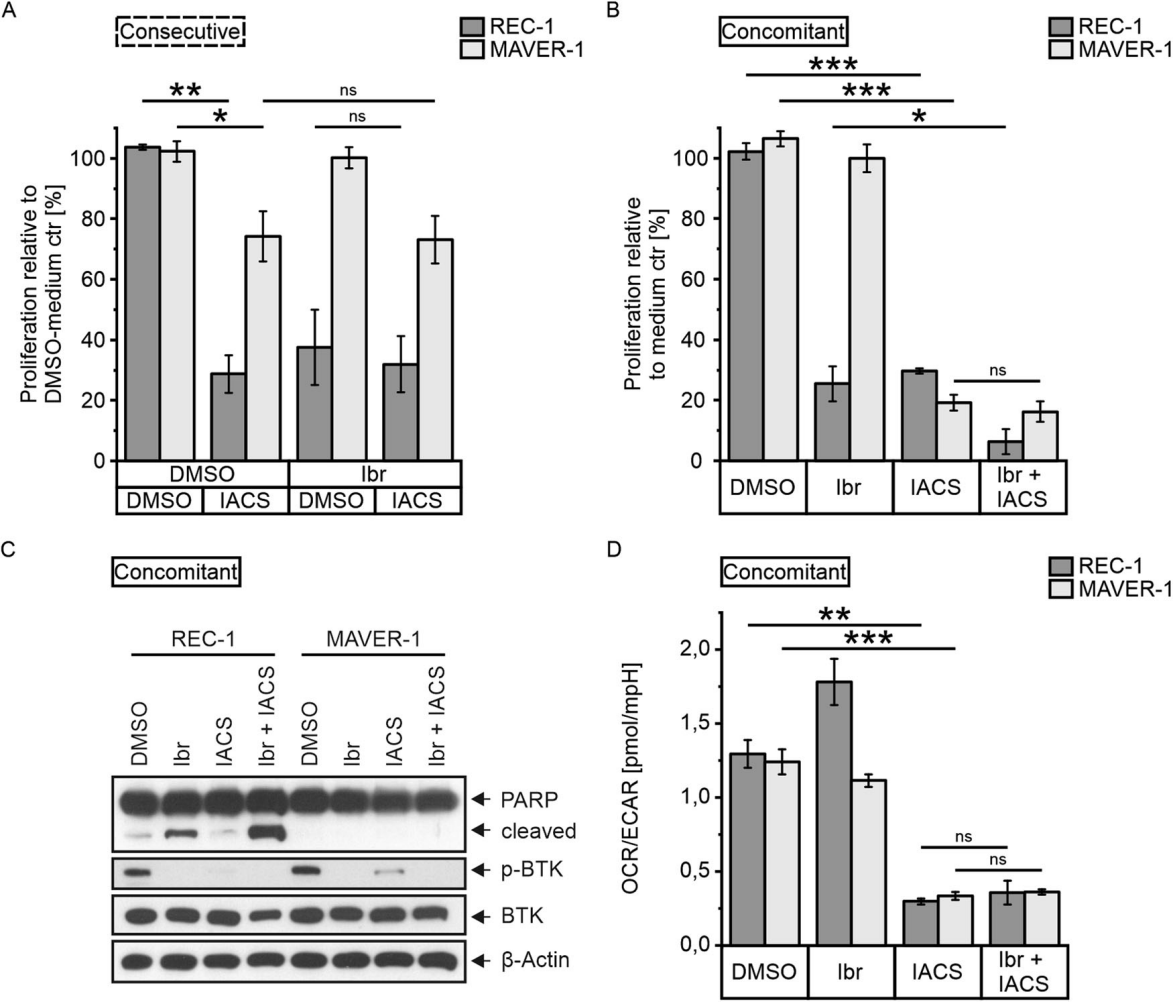
Combination of ibrutinib and IACS-010759 treatment. **A** Consecutive treatment of REC-1 and MAVER-1 with 3 d ibrutinib pretreatment (400 nM or DMSO), followed by incubation of viable cells with 25 nM IACS-010759 or DMSO and 400 nM ibrutinib or DMSO according to pretreatment for 2 more days, and **B** Concomitant ibrutinib (400 nM) plus IACS-010759 (25 nM) treatment of REC-1 and MAVER-1 across 4 d; proliferation was assessed by 3-(4,5-dimethylthiazol-2-yl)-2,5-diphenyltetrazolium bromide (MTT) assay and percentages relative to medium control (ctr) pretreated with DMSO (**A**; *N* = 3) and relative to medium control (**B**; *N* = 4) are shown. **C** Western blot with PARP, p-BTK, BTK, and β-Actin as loading control (representative for *N* = 3; see supplementary Original Western Blots), and **D** Extracellular flux analysis showing oxygen consumption rate vs extracellular acidification rate ratios (*N* = 4) of 3 d simultaneous treatment with 400 nM ibrutinib and 25 nM IACS-010759. Data are shown as mean ± SEM. Significance was determined by two-sided Student’s *t*-test or a Welch’s *t*-test, for equal or unequal variances, respectively. *P < 0.05, **P < 0.01, ***P < 0.001, *P* > 0.05 not significant (ns). Significance is indicated for relevant comparisons.

Cells were first treated with 400 nM ibrutinib for 3 d to trigger reliance on OXPHOS, before 25 nM IACS was added to the surviving cells for 2 additional days. In the DMSO-pretreated controls, OXPHOS inhibition caused stronger decrease in proliferation in REC-1 (DMSO/DMSO vs DMSO/IACS, *P* = 0.007) than in the ibrutinib-resistant control cell line MAVER-1 (DMSO/DMSO vs DMSO/IACS, *P* = 0.035; Fig. 2A). However, the two cell lines showed a differential response over time. The kinetic course of proliferation revealed higher proliferation loss in REC-1 at 1–3 d treatment with IACS compared with MAVER-1 (Supplementary Fig. 2A). This changed at 4 d treatment, as MAVER-1 showed a sharp decrease in proliferation, whereas the proliferation of REC-1 remained constant over 3–4 d treatment. In consecutive treatment, MAVER-1 was not affected by ibrutinib treatment and proliferation remained unchanged between IACS only treatment and the combination with ibrutinib (ibrutinib/DMSO vs ibrutinib/IACS, *P* = 0.927; Fig. 2A). In REC-1, ibrutinib alone resulted in a decrease in proliferation to 37%. However, the addition of IACS to ibrutinib did not further reduce proliferation (ibrutinib/DMSO vs ibrutinib/IACS, *P* = 0.789; Fig. 2A), increase apoptosis or reduce OCR/ECAR ratios (Supplementary Fig. 2B and C, supplementary Original Western Blots).

Given the poor effect of IACS in consecutive regimen, we tested whether concomitant use of ibrutinib and IACS for 04 d would have a superior effect to ibrutinib treatment alone. In REC-1, a significant decrease in proliferation was observed, from 25% after ibrutinib alone to 6% in combination with IACS (ibrutinib vs ibrutinib + IACS, *P* = 0.035; Fig. 2B). This was accompanied by increased apoptosis as indicated by higher PARP cleavage after 03 d treatment (Fig. 2C, supplementary Original Western Blots). In MAVER-1, IACS impaired proliferation (DMSO vs IACS, *P* < 0.001) and the effect of the combination was similar to IACS only treatment (IACS vs ibrutinib + IACS, *P* = 0.500), as ibrutinib had no effect (Fig. 2B). Loss in proliferation due to 04 d of IACS was higher in MAVER-01 than in REC-01 (DMSO vs IACS, *P* < 0.001 for REC-1), which was opposite to 02 d treatment described above and reflected the proliferation course of the two cell lines across IACS treatment, as shown by the kinetics (Supplementary Fig. 2A). As PARP was not inactivated following 03 d IACS only treatment in both cell lines, reduced proliferation was not associated with increased apoptosis (Fig. 2C, supplementary Original Western Blots). In contrast, IACS affected activation of BTK, resulting in reduced (MAVER-1) or no phosphorylation (REC-1). The extracellular flux analysis revealed low oxygen consumption rate (OCR) vs extracellular acidification rate (ECAR) ratios in both cell lines following 03 d incubation with IACS (DMSO vs IACS, *P* = 0.002 for REC-01 and *P* < 0.001 for MAVER-1; Fig. 2D). Even though ibrutinib caused an increased OCR/ECAR ratio in REC-01 cells, as previously reported by Fuhr et al. [7], IACS led to similarly low OCR/ECAR ratios in REC-01 and MAVER-01 independent of the combination with ibrutinib (IACS vs ibrutinib + IACS, *P* = 0.525 for REC-01 and *P* = 0.467 for MAVER-1).

### Ibrutinib-sensitive cells are highly susceptible to CD52 mAb-mediated complement-dependent cytotoxicity following ibrutinib pretreatment

**Fig. 3:**
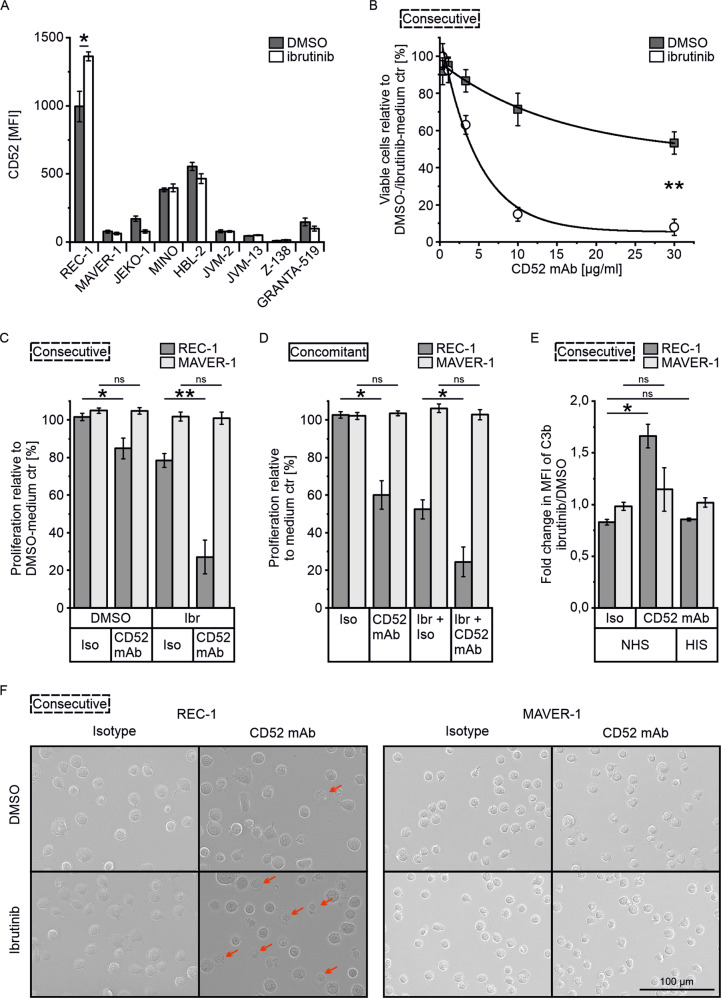
Combination of ibrutinib and CD52 mAb with human serum. **A** CD52 surface levels of the indicated mantle cell lymphoma cell lines as mean fluorescence intensity (MFI, median) after 3 d treatment with 400 nM ibrutinib (or DMSO) analyzed by flow cytometry using CD52-PE (*N* = 3). **B** Titration of CD52 mAb concentration in combination with 10% normal human serum (NHS) on surviving cells of 3 d ibrutinib (400 nM) or DMSO pretreatment (consecutive treatment); viable cells (PI-) were determined by flow cytometry after 30 min of serum addition and percentage is shown compared to medium control (ctr) of ibrutinib- or DMSO-pretreated cells (*N* = 3). **C** Consecutive treatment as described in (**B**) with 10 µg/ml CD52 mAb (or isotype) and 10% NHS for 15 min, and **D** Concomitant treatment with ibrutinib plus 10 µg/ml CD52 mAb (or isotype) and 10% NHS across 48 h; proliferation was determined by 3-(4,5-dimethylthiazol-2-yl)-2,5-diphenyltetrazolium bromide (MTT) assay and is shown as a percentage compared to medium control of ibrutinib- or DMSO-pretreated cells (**C**, *N* = 4), and medium control (**D**, *N* = 3). **E** C3b deposition on cell surface following consecutive treatment as described in (**C**) using heat inactivated human serum (HIS) as control; fold change in C3b-FITC mean fluorescence intensity (MFI, geometric mean) between ibrutinib- and DMSO-pretreated cells as determined by flow cytometry (*N* = 3). **F** Microscopy images following consecutive treatment as described in (**C**), representative for three independent experiments. Data are shown as mean ± SEM. Significance was determined by two-sided Student’s *t*-test or a Welch’s *t*-test, for equal or unequal variances, respectively. *P < 0.05, **P < 0.01, *P* > 0.05 not significant (ns). Significance is indicated for relevant comparisons.

The CD52 surface expression of 8 MCL cell lines was determined after 3 d ibrutinib (400 nM) incubation to check whether any responded similarly to REC-1 with increased expression. REC-1 had the highest basal CD52 level and was the only cell line upregulating CD52 surface expression with ibrutinib (*P* = 0.035, Fig. 3A). Therefore, subsequent experiments studied CD52 mAb therapy in combination with human serum as a source of complement only in REC-1 cells following 3 d ibrutinib pretreatment (consecutive setup) and included MAVER-1 as ibrutinib-resistant MCL cell line.

Different antibody concentrations were tested on ibrutinib- or DMSO-pretreated REC-1 cells. The ibrutinib group showed higher sensitivity than the DMSO group, with 8 and 53% remaining viable cells at 30 µg/ml after only 30 min of serum incubation, respectively (DMSO/CD52 mAb vs ibrutinib/CD52 mAb, *P* = 0.004; Fig. 3B). The maximum effect on viability was reached at a minimum antibody concentration of 10 µg/ml, that was used for the following experiments.

Since dead cells from pretreatment were removed and only viable REC-1 cells were tested in consecutive setup, proliferation of REC-1 cells was still 78% in ibrutinib-pretreated isotype control (Fig. 3C). Upon addition of CD52 mAb with NHS, proliferation of ibrutinib-pretreated REC-1 dropped to 27% (ibrutinib/isotype vs ibrutinib/CD52 mAb, *P* = 0.002), whereas DMSO group still showed 85% of proliferation (DMSO/isotype vs DMSO/CD52 mAb, *P* = 0.030). MAVER-1 cells were neither affected by ibrutinib nor by anti-CD52 (DMSO/isotype vs DMSO/CD52 mAb, *P* = 0.927 and ibrutinib/isotype vs ibrutinib/CD52 mAb, *P* = 0.831).

With respect to the higher efficacy of concomitant use of ibrutinib and IACS, the simultaneous treatment with ibrutinib, CD52 mAb, and NHS for 48 h was investigated. CD52 mAb treatment alone led to a decrease in proliferation to 60% in REC-1 (isotype vs CD52 mAb, *P* = 0.032; Fig. 3D). Concomitant ibrutinib plus CD52 mAb resulted in a significant decrease in proliferation to 24% compared to 52% with ibrutinib plus isotype (*P* = 0.040). Again, proliferation of MAVER-1 remained unchanged across 48 h treatment with ibrutinib, CD52 mAb (isotype vs CD52 mAb, *P* = 0.553), and the combination (ibrutinib + isotype vs ibrutinib + CD52 mAb, *P* = 0.397).

Relative to the corresponding ibrutinib/isotype control, decrease of proliferation was 28% for concomitant incubation, and 51% for consecutive treatment. Due to the higher and fast toxicity of the consecutive approach on ibrutinib-sensitive REC-1, next experiments were performed accordingly.

To investigate whether CD52 mAb mediated toxicity was caused by complement activation, the deposition of the complement component C3b on cell surface was tracked by flow cytometry. Upon addition of CD52 mAb and NHS, the C3b level on cell surface of ibrutinib-pretreated REC-1 was 1.7 fold higher than in the DMSO-pretreated control (Fig. 3E). The fold change of C3b of CD52 mAb between ibrutinib and DMSO pretreatment was significantly higher compared to isotype control (*P* = 0.019). When heat inactivated human serum (HIS) was added instead of NHS, no complement activation was determined and no difference between ibrutinib and DMSO pretreatment was observed (isotype/NHS vs CD52 mAb/HIS, *P* = 0.442). In MAVER-1, the addition of CD52 mAb or isotype with NHS or HIS did not induce significant differences of C3b deposition (isotype/NHS vs CD52 mAb/NHS, *P* = 0.526) and ibrutinib pretreatment did not show an effect on extent of complement activation compared to DMSO control.

Microscopy showed cell lysis indicated by cell swelling and bloated plasma membranes in REC-1 cells treated with CD52 mAb and NHS (Fig. 3F). The extent of cell lysis was the highest following ibrutinib pretreatment. In contrast, the morphology of MAVER-1 cells was not affected by the antibody.

### Ibrutinib triggers upregulation of CD52 in a subgroup of MCL patients and causes higher susceptibility to CD52 mAb mediated cytotoxicity in ibrutinib-sensitive cases

Primary mantle cell lymphoma cells collected from patients at first diagnosis and from one patient after relapse (P3) were included to study the effect of treatment with ibrutinib and CD52 mAb (8 peripheral blood mononuclear cell (PBMC), 2 lymph node, and 2 healthy control PBMC samples; see Supplementary Table 2 for patient characteristics). As the previous experiments revealed higher toxicity of the consecutive use of ibrutinib and CD52 mAb, cells were first treated for 2–3 d with 400 nM ibrutinib depending on cell fitness.

**Fig. 4:**
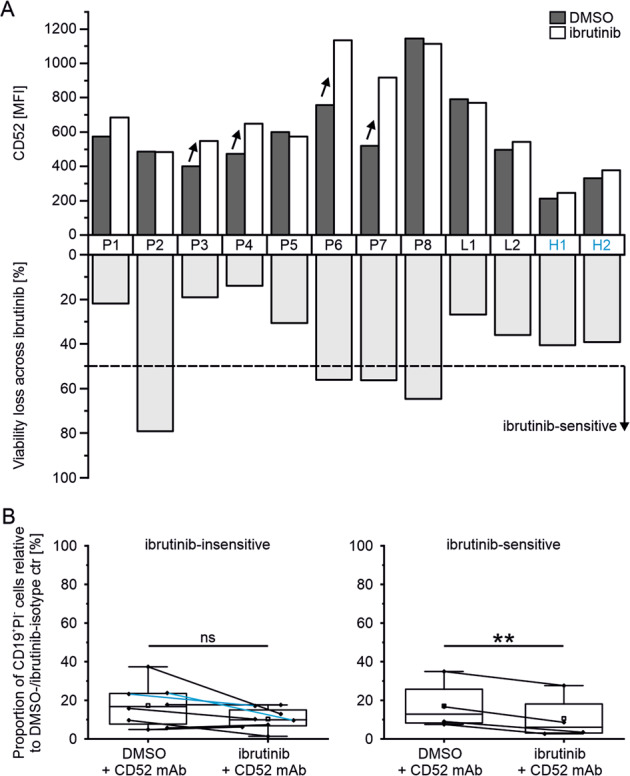
CD52 as target in ibrutinib-pretreated primary MCL cells. **A** CD52 levels of primary CD19^+^PI^−^ cells from peripheral blood mononuclear cells (PBMCs, P) and lymph nodes (L) from mantle cell lymphoma patients and PBMCs from healthy controls (H, blue) as mean fluorescence intensity (MFI, median) after 2–3 d treatment (patient characteristics in Supplementary Table 2) with ibrutinib (400 nM, DMSO as control), and percentage loss of CD19^+^PI^−^ cells after ibrutinib incubation compared with DMSO control (below); data were acquired by flow cytometry using CD52-APC, cell debris and doublets were excluded. B Effect of 10 µg/ml CD52 mAb treatment in combination with 10% normal human serum (NHS) on viability of ibrutinib- or DMSO-pretreated primary MCL cells; boxplot (centerline as median and square as mean) shows the percentage of CD19^+^PI^−^ cells after treatment with CD52 mAb in ibrutinib-insensitive (viability loss by ibrutinib < 50%, left) and ibrutinib-sensitive (viability loss by ibrutinib > 50%, right) cases relative to control treatment with isotype control of the corresponding pretreatment (ctr); Shapiro-Wilk test was performed to test the data for normal distribution and significance was determined by Paired *t*-test, **P < 0.01, *P* > 0.05 not significant (ns).

Given the wide variation in response to ibrutinib, cases with viability loss >50% were considered as ibrutinib-sensitive (P2, P6, P7, P8) and those with <50% as ibrutinib-insensitive (Fig. 4A). The surface antigen CD52 was expressed in all included primary MCL cells and the CD52 level on the tumor cells was higher compared to healthy control B cells (H1, H2). In 4 out of 10 MCL patients, CD52 surface expression increased by more than 25% with ibrutinib. Two of these cases belonged to ibrutinib-sensitive (P6, P7) and two to ibrutinib-insensitive cohort (P3, P4). Subsequent treatment with 10 µg/ml CD52 mAb and 10% NHS strongly reduced viability of malignant and healthy B cells (Fig. 4B). However, significantly lower viability of the ibrutinib- compared to DMSO-pretreated cells was observed in the ibrutinib-sensitive cohort following anti-CD52 therapy (*P* = 0.003). Specifically, in two (P6, P7) of 10 primary samples, ibrutinib led to reduced viability of more than 50%, triggered considerable upregulation of surface CD52, and rendered the tumor cells more vulnerable to CD52 mAb mediated toxicity, as seen in the ibrutinib-sensitive cell line model.

## Discussion

As resistance to ibrutinib therapy in MCL is common, its combination with other agents may reduce the risk for relapse [38]. Recently, multiple trials tested the efficacy of ibrutinib in combination with anti-CD20 monoclonal antibodies rituximab or obinutuzumab, and the small molecules venetoclax, bendamustine, and lenalidomide [39–41]. Although their anti-tumor activity is complementary to ibrutinib, a drug that overcomes specifically acquired ibrutinib resistance may be a superior partner. Some mechanisms of resistance were identified in the last years, such as mutation of the BTK at ibrutinib’s binding site, circumvention by alternative signaling pathways, protective microenvironment, and metabolic reprogramming towards addiction to OXPHOS, but they only cover a small part of the spectrum of resistance [19, 42–44].

In a prior scRNA-seq analysis, we tracked an ibrutinib-sensitive MCL cell line across 2 d treatment with ibrutinib, as adaptations in gene expression could be associated with higher resistance to the BTK inhibitor [7]. Although the four surviving subpopulations showed a common response, the existence of two metabolic distinct subgroups indicated that we had not caught the finally resistant cells. However, the here performed RNA-seq study demonstrated, that the transcriptome of the ibrutinib-sensitive REC-1 did not change considerably across 2–4 d treatment. Nevertheless, pathway analysis revealed reduced glycolysis and increased activity of OXPHOS in resisting cells after 4 d exposure supporting the hypothesized higher dependence on oxidative phosphorylation triggered by ibrutinib in sensitive cells [7].

Besides basal ATP generation by OXPHOS, glycolysis is a less efficient source for energy, but provides the precursors for the synthesis of biomass required for accelerated tumor cell growth [45]. The balance between OXPHOS and glycolysis-based metabolism varies in cancer cells and can be addressed by extracellular flux analysis. In contrast to the observations by Zhang et al., the differences in OCR/ECAR ratios between ibrutinib-sensitive and resistant MCL cell lines were not confirmed by our results, showing similar ratios for ibrutinib-sensitive REC-1 and resistant MAVER-1, which may be due to different culture conditions, cell density, and experimental setup in general [19, 46, 47]. In view of the impairment of glycolytic capacity by ibrutinib and the triggering of OXPHOS dependence in surviving MCL cells, the OXPHOS inhibitor IACS-010759 was added to ibrutinib-pretreated cells to block the metabolic escape [7, 18]. Surprisingly, the sequential combination did not reduce proliferation and viability of the tumor cells compared to ibrutinib alone, as cells likely compensated quenched OXPHOS by restoring glycolysis [48]. In contrast, simultaneous ibrutinib treatment plus OXPHOS inhibition increased toxicity. However, for in vivo use, an acceptable side effect profile must be ensured with respect to the proliferation already heavily reduced by the single agents. Considering the interplay between growth stimulating signaling pathways such as BTK signaling and bioenergetic processes in malignant B cells, combination regimens have to be carefully evaluated to avoid cross-reducing effects and thus maximize their efficacy [49]. Patients may also profit from IACS-010759 as a single agent, since its anti-proliferative effects on ibrutinib-sensitive and resistant MCL cells may specifically mitigate fast proliferating cells and reduce tumor burden. This study, in correlation with Zhang et al., highlights the use of OXPHOS inhibition in ibrutinib-treated mantle cell lymphoma cells [19]. However, further studies need to decipher the benefits in patient subgroups.

Inhibition of BCR signaling by ibrutinib resulted in decreased signaling of associated pathways (NF-κB, PI3K-Akt, TNF, Toll-like receptor) and lower cell cycle activity. Besides, loss of cell adhesion signature might be linked to the impaired BCR signaling and associated with the reported higher levels of CD52 with potential anti-adhesion property [7, 28, 29, 50]. Redistribution of MCL cells from lymph nodes into peripheral blood is frequent with ibrutinib therapy, and the detachment may coincide with increased CD52 surface expression [51].

Use of anti-CD52 antibody in a consecutive setup resulted in fast depletion of ibrutinib surviving REC-1 cells by complement-dependent cytotoxicity. The results encouraged us to evaluate alterations of CD52 levels and to test the efficacy of anti-CD52 therapy on primary MCL cells after pretreatment with ibrutinib. The BTK inhibitor affected healthy and malignant B cells, but only four of the primary MCL cases showed substantial loss of viability (ibrutinib-sensitive). Clinical studies reported 68–69% overall response to ibrutinib, which contrasts to the here observed 40% in untreated primary MCL cells [52, 53]. Cryopreservation and culturing without stimulating agents may have limited the signaling activity of MCL cells and responsiveness to ibrutinib. However, standard primary B-cell cultivation protocols were not appropriate, as these would alter signaling and thereby abrogate the effect of ibrutinib [43]. Focusing on the primary ibrutinib-sensitive samples revealed that ibrutinib pretreatment rendered the MCL cells significantly more vulnerable to anti-CD52 toxicity, although only two had increased CD52 levels. In summary, 20% of the 10 included primary MCL samples reflected the behavior of the ibrutinib-sensitive cell line model REC-1. The low rate might be due to limited number of samples, but also indicates that only a subgroup of MCL patients would potentially profit from the anti-CD52 treatment approach. Flow cytometry analysis of blood samples from ibrutinib responders may detect cases with increased CD52 antigen density on MCL cells, which may be more prone to CD52 mAb-mediated CDC [54]. Since ibrutinib induces lymphocytosis, residual MCL cells in the blood could be cleared by alemtuzumab, as the anti-CD52 antibody is more active in blood than in lymph nodes and could help to yield MRD negativity [55]. However, the observed fast depletion of healthy lymphocytes is consistent with the known serious side-effect profile of the antibody, including the risk of immunosuppression and viral and opportunistic infections, which must be taken into account in clinical use and may limit its applicability to heavily pretreated MCL patients with poor prognosis [56].

Further studies need to corroborate the results with the clinically used humanized IgG1 kappa antibody alemtuzumab, since antibodies of the IgG1 subclass induce higher complement activation than the IgG2 used in this study [57]. Moreover, it is of interest, whether the here shown CDC in REC-1 or another mechanism like ADCC is involved in the in vivo performance of anti-CD52 treatment in MCL [58].

In conclusion, OXPHOS and CD52 were identified as features of MCL cells that resisted ibrutinib therapy. Preclinical evaluation of two potential agents for these targets emphasized the higher efficacy of anti-CD52 therapy as consolidation after prior ibrutinib treatment, potentially minimizing survival of residual, resistant cells and improving prognosis of MCL patients.

## Materials and methods

### Cell lines, reagents, and antibodies

The cell lines REC-1, MAVER-1, JEKO-1, MINO, GRANTA-519, JVM-2, and JVM-13 were from the Deutsche Sammlung von Mikroorganismen und Zellkulturen (DSMZ, Braunschweig, Germany). Z-138 was available from the Lymphoma Research Foundation and was obtained from the American Type Culture Collection (ATCC, Manassas, VA, USA). HBL-2 was provided by Florian Bassermann (Department of Medicine III, Technische Universität München, Munich, Germany). Cell lines were handled according to master and working stock system, and cells were cultured as previously described [59]. Mycoplasma contamination was ruled out applying the Venor® GeM OneStep kit.

Reagents and antibodies are listed in supplementary information.

### Bulk RNA-sequencing

Cells were either untreated or treated with 400 nM ibrutinib for 2 d, 3 d, and 4 d. After 4 d incubation, viable cells were collected (see viable cell isolation) and washed with PBS before isolating the RNA using AllPrep DNA/RNA Mini Kit.

RNA quality was checked using a 2100 Bioanalyzer with the RNA 6000 Nano Kit. DNA libraries were prepared using the TruSeq Stranded mRNA Library Preparation Kit according to manufacturer’s instructions (1/2 volume). Sequencing of pooled libraries was performed in single-end mode with 75 nt read length on the NextSeq 500 platform (Illumina, San Diego, CA, USA) using High output sequencing kits. Demultiplexed FASTQ files were generated with bcl2fastq2 v2.20.0.422 (Illumina). To assure high sequence quality, Illumina reads were quality- and adapter-trimmed via Cutadapt v.2.5 using a cutoff Phred score of 20 in NextSeq mode, and reads without any remaining bases were discarded [60]. Processed reads were subsequently mapped to the human reference genome (Ensembl GRCh38) using STAR v.2.7.2b [61]. Read counts on exon level summarized for each gene were generated using featureCounts v.1.6.4 from the Subread package [62]. The count output was utilized to identify differentially expressed genes using DESeq2 v.1.24.0 [63]. Read counts were normalized by DESeq2 and fold-change shrinkage was applied by setting the parameter “betaPrior=TRUE”. Differential expression of genes was assumed at an adjusted *P*-value (padj) after Benjamini-Hochberg correction < 0.05 and |log2FoldChange| ≥ 1.

ClusterProfiler v.3.14.3 was used for gene set enrichment analysis (GSEA) on all detected differentially expressed genes (DEGs) ranked by their DESeq2 log2FoldChange (log2FC) [64].

### Viable cell isolation

Cells were resuspended in density gradient medium OptiPrep^TM^ diluted with growth medium (14% (w/v) iodixanol) and overlaid with phosphate buffered saline (PBS). Viable cells were aspirated from the interphase after centrifugation. High viability (≥87% viable cells) was verified by a Countess® II FL Automated Cell Counter (Thermo Fisher Scientific, Waltham, MA, USA) following trypan blue staining of cells.

### Proliferation assay

Metabolic activity of cells as a measure of proliferation was determined by 3-(4,5-dimethylthiazol-2-yl)-2,5-diphenyltetrazolium bromide (MTT) assay as previously published [43].

### Western Blot

After incubation of cells as indicated, protein lysates were prepared and western blot was performed as previously described [59].

### Extracellular flux analysis

Cells were resuspended in Seahorse medium (Agilent Technologies, Santa Clara, CA, USA) and were seeded in poly-D-lysine coated Seahorse XFe96 Cell Culture Microplates (Agilent). Extracellular Flux analysis was performed on a Seahorse XFe96 Metabolic Flux Analyzer (Agilent) as described earlier [7].

### CD52 mAb and IACS-010759 treatment

Cells were either pretreated (with subsequent isolation of viable cells, except for Supplementary Fig. 2C) or simultaneously treated with 400 nM ibrutinib or DMSO and 10 µg/ml CD52 mAb (or isotype control) for anti-CD52 assay, or 25 nM IACS-010759 (or DMSO) for OXPHOS inhibition. For anti-CD52 assay, cells were seeded in serum-free medium on ice and incubated with CD52 mAb (or isotype control) for 30 min, before normal human serum (NHS, 10%) was added as source for active complement (heat inactivated human serum as control, HIS) for further incubation at 37 °C and 5% CO_2_ in humidified atmosphere.

### Microscopy

Images were taken with an EVOS M7000 Imaging System (Thermo Fisher Scientific, Waltham, MA, USA) and converted to grayscale (8-bit) by Corel PHOTO-PAINT 2019 (Ottawa, ON, Canada).

### Primary cells

Primary cells, deposited in the Interdisciplinary Bank of Biomaterials and Data were used according to the ethical guidelines of the Medical Faculty. After isolation of cells from lymph nodes using cell sieves and from blood by Ficoll, cells were frozen and stored in liquid nitrogen. Culture medium consisted of RPMI 1640, 2 mM L-Glutamine, 10% HIS, 1% penicillin-streptomycin, and 1% non-essential amino acids (100x MEM NEAA).

### Flow cytometry

Data were acquired on a BD FACSCanto II (BD Bioscience, CA, USA) with FACS Diva Software v.6.1.3 and was analyzed with Flowing Software v.2.5.1 (Perttu Terho, Turku Center for Biotechnology, University of Turku, Finland, in collaboration with Turku Bioimaging). Cells were stained with propidium iodide (PI) to identify viable cells. Isotype controls were included to exclude unspecific antibody binding.

### Statistical analysis

Statistics were prepared in Microsoft Excel 2016 (Microsoft Corporation, Redmond, WA, USA) and OriginPro® 2021b (OriginLab Corporation, Northampton, MA, USA). Statistical analyses are described in the figure legends. Groups were considered significantly different when *P* < 0.05 (*), *P* < 0.01 (**), *P* < 0.001 (***) or not significant (ns) at *P* > 0.05. *P*-values are mentioned for decisive results of this study. Figures show mean values ± standard error of the mean (SEM).

## Supplementary information

Supplementary Information

Supplementary Table 1

Original Western Blots

## Data Availability

The RNA-seq data set is available in the Gene Expression Omnibus repository under GEO accession GSE214725.
