# Supplementary material for: CD52 and OXPHOS—potential targets in ibrutinib-treated mantle cell lymphoma
Source: Cell Death Discov. 2022 Dec 31;8:505. doi: 10.1038/s41420-022-01289-7 (PMC9805448; doi:10.1038/s41420-022-01289-7)
Supplement: Supplementary file 1 — Supplementary Information [file 41420_2022_1289_MOESM1_ESM.pdf]

Supplementary Information

Contents:

- **Reagents and antibodies**
- **Supplementary Table 1:** Enriched KEGG pathways of 4 d ibrutinib-treated REC-1
- **Supplementary Table 2:** Characteristics of primary samples
- **Supplementary Figure 1:** Enrichment plots for selected KEGG pathways of 4 d ibrutinib-treated REC-1
- **Supplementary Figure 2:** IACS-010759 kinetics and consecutive treatment with ibrutinib and IACS-010759

Reagents and antibodies

Reagents

Venor® GeM OneStep kit	Minerva Biolabs, Berlin, Germany
AllPrep DNA/RNA Mini Kit	Qiagen, Hilden, Germany
RNA 6000 Nano Kit	Agilent Technologies, Santa Clara, CA, USA
TruSeq Stranded mRNA Library Preparation Kit	Illumina, San Diego, CA, USA
OptiPrep™	Serumwerk Bernburg, Bernburg, Germany
Ficoll® Paque Plus	Cytiva, Marlborough, MA, USA
RPMI 1640	Thermo Fisher Scientific, Waltham, MA, USA
L-Glutamine	PAN-Biotech, Aidenbach, Germany
Penicillin-streptomycin	PAN-Biotech
100x MEM NEAA	PAN-Biotech
Propidium iodide	Sigma-Aldrich, St. Louis, MO, USA

Reagents - inhibitors

Ibrutinib	Selleck Chemicals, Absource Diagnostics, Munich, Germany
IACS-010759	Selleck Chemicals

Antibodies – cytotoxicity assays

CD52 mAb (#MA5-16999)	Invitrogen, Thermo Fisher Scientific
Rat IgG2bκ Isotype Control (#16-4031-81)	eBioscience™, Thermo Fisher Scientific

Antibodies – western blots

PARP (#9532)	Cell Signaling Technologies, Danvers, MA, USA
p-BTK (#5082)	Cell Signaling Technologies
BTK (#3533)	Cell Signaling Technologies
β-Actin (#4970)	Cell Signaling Technologies
Anti-rabbit IgG, HRP-linked Antibody (#7074)	Cell Signaling Technologies

Antibodies – flow cytometry

CD19-FITC (#FAB4867F)	R&D Systems, Minneapolis, MN, USA
C3b/iC3b-FITC (#846108)	BioLegend, San Diego, CA, USA
Mouse IgG1κ (#IC002F)	R&D Systems
CD52-PE (#130-123-972)	Miltenyi Biotech, Bergisch Gladbach, Germany
REA Control (S) PE (#130-113-438)	Miltenyi Biotech
CD52-APC (#130-099-632)	Miltenyi Biotech
REA Control (S) APC (#130-113-434)	Miltenyi Biotech

Supplementary Table 1: Enriched KEGG pathways of 4 d ibrutinib-treated REC-1

For gene sets with |log2FC| ≥ 1 and q value < 0.05 of Kyoto Encyclopedia of Genes and Genomes (KEGG) pathway gene set enrichment analysis (GSEA) of 4 d ibrutinib treated REC-1 (versus untreated) see excel file of Supplementary Table 1.

Supplementary Table 2: Characteristics of primary samples

Sample	Type	Sex	Mantle cell lymphoma subtype	Therapy prior to sample collection	In vitro (treatment)
P1	PBMC	m	classic nodal	no	3 d
P2	PBMC	m	classic nodal	no	3 d
P3	PBMC	m	classic nodal	yes, Nordic protocol, ibrutinib	2 d
P4	PBMC	m	classic nodal	no	2 d
P5	PBMC	f	leukaemic non-nodal	no	3 d
P6	PBMC	m	classic nodal	no	3 d
P7	PBMC	m	classic nodal	no	3 d
P8	PBMC	m	classic nodal	no	3 d
L1	Lymph node	m	classic nodal	n.a., pre-ibrutinib era	2 d
L2	Lymph node	m	classic nodal	n.a., pre-ibrutinib era	2 d
H1	PBMC	f	healthy control	no	2 d
H2	PBMC	f	healthy control	no	3 d

PBMC = peripheral blood mononuclear cells, m = male, f = female, d = days

Supplementary Figure 1

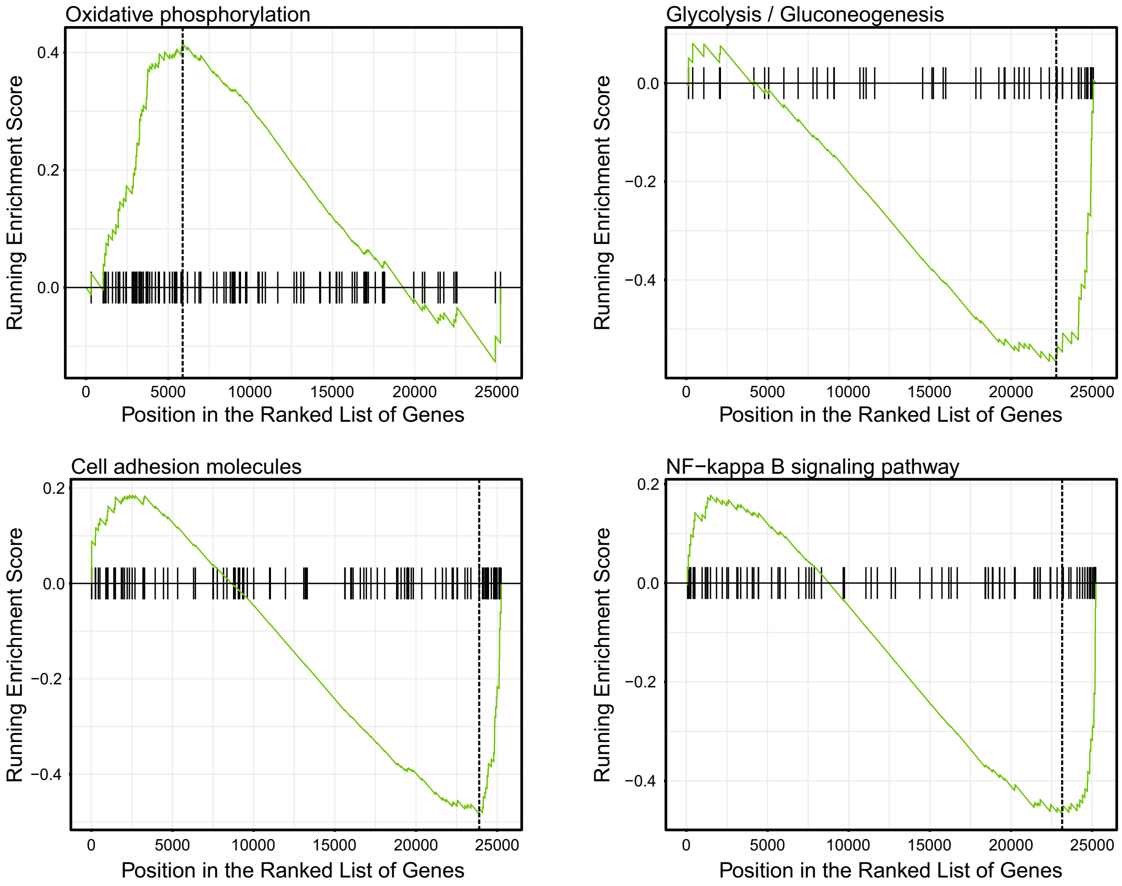

Supplementary Figure 1: Enrichment plots for selected KEGG pathways of 4 d ibrutinib-treated REC-1.

Enrichments plots of selected significant gene sets by the Kyoto Encyclopedia of Genes and Genomes (KEGG) pathway gene set enrichment analysis (GSEA) of 4 d ibrutinib treated REC-1 compared with untreated (|log2FC| ≥ 1, q value < 0.05).

Supplementary Figure 2

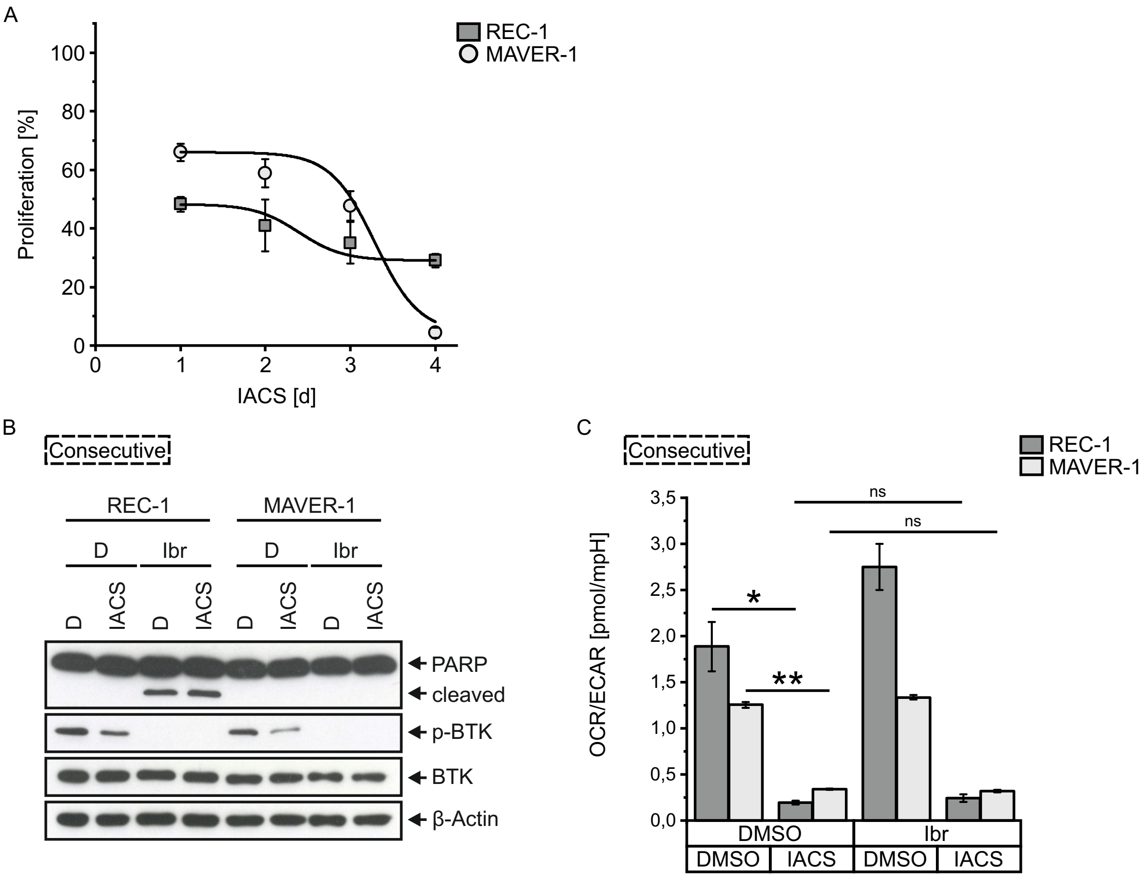

Supplementary Figure 2: IACS-010759 kinetics and consecutive treatment with ibrutinib and IACS-010759.

A) Proliferation of REC-1 and MAVER-1 across 1 to 4 d treatment with 25 nM IACS-010759, determined by 3-(4,5-dimethylthiazol-2-yl)-2,5-diphenyltetrazolium bromide (MTT) assay (N = 3). B) Western blot with PARP, p-BTK, BTK, and β-Actin as loading control (representative for N = 3), after consecutive treatment with 3 d ibrutinib pretreatment (400 nM, DMSO as control) followed by isolation and incubation of viable cells with 25 nM IACS-010759 or DMSO and 400 nM ibrutinib or DMSO according to pretreatment for 2 more days. C) Extracellular Flux analysis showing oxygen consumption rate (OCR) vs extracellular acidification rate (ECAR) ratios of REC-1 and MAVER-1 after consecutive treatment with 3 d ibrutinib pretreatment (400 nM, DMSO as control) followed by incubation with 25 nM IACS-010759 or DMSO and 400 nM ibrutinib or DMSO according to pretreatment for 2 more days (N = 4) .

Data is shown as mean ± SEM. Significance was determined by Student's t-test or a Welch's t-test, for equal or unequal variances, respectively. * $P < 0.05$, ** $P < 0.01$, $P > 0.05$ not significant (ns).
