# Supplementary material for: CD52 and OXPHOS—potential targets in ibrutinib-treated mantle cell lymphoma
Source: Cell Death Discov. 2022 Dec 31;8:505. doi: 10.1038/s41420-022-01289-7 (PMC9805448; doi:10.1038/s41420-022-01289-7)

Original western blots

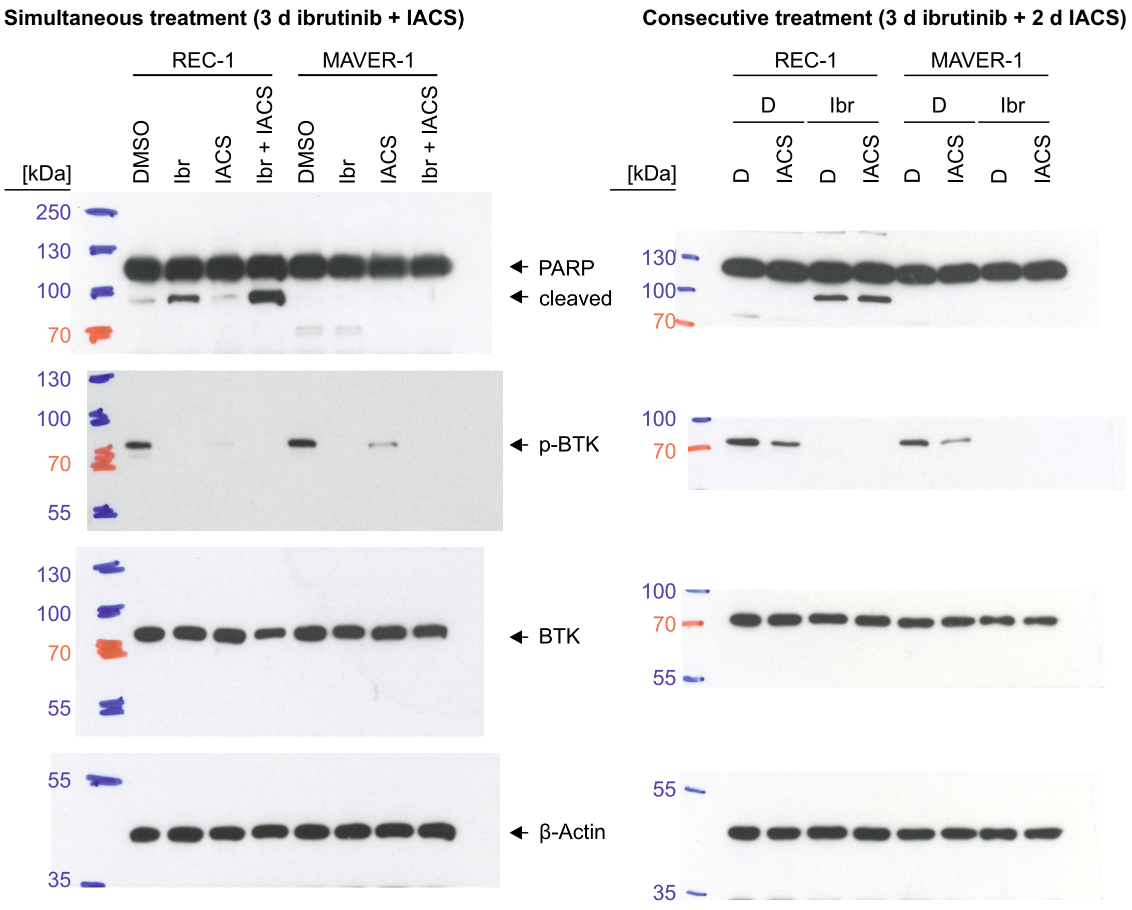

Supplement: Supplementary file 3 — Original Western Blots [file 41420_2022_1289_MOESM3_ESM.pdf]
